# Lifesaving Thoracic Endovascular Aortic Repair for Traumatic Aortic Isthmus Rupture: A Case Report

**DOI:** 10.7759/cureus.82048

**Published:** 2025-04-10

**Authors:** Athanasios Papatriantafyllou, Konstantina Soukouli, Paraskevi Dedopoulou, Vasileios Leivaditis, Ioannis Karioris, Michail Peroulis, Manfred Dahm, Stylianos Tsochatzis

**Affiliations:** 1 Department of Cardiothoracic and Vascular Surgery, Westpfalz-Klinikum, Kaiserslautern, DEU; 2 Department of Surgery, General Hospital of Patras, Patras, GRC; 3 Vascular Unit, Department of Surgery, Medical School, University of Ioannina, Ioannina, GRC

**Keywords:** aortic isthmus, aortic rupture, blunt thoracic aortic injury, endovascular repair, thoracic endovascular aortic repair

## Abstract

Blunt thoracic aortic injury (BTAI) is a high-energy trauma, with most patients succumbing at the scene of the accident. Those who reach the hospital require immediate intervention. In most cases, the injury affects the aortic isthmus, and endovascular repair is the typical treatment approach. We present a rare case of BTAI in a 68-year-old female patient who arrived at the ED following a pedestrian accident. CT imaging revealed a grade III BTAI, which was successfully treated with thoracic endovascular aortic repair. This case underscores the critical importance of timely diagnosis and prompt intervention in BTAI.

## Introduction

Trauma continues to be the leading cause of death among young adults, with traumatic brain injuries at the forefront, followed by blunt thoracic aortic injury (BTAI). These injuries typically result from high-energy mechanisms, most commonly related to traffic accidents. Approximately 80% of patients succumb before reaching the hospital, and around one in two patients die during hospitalization. This rare type of injury accounts for 1.5% of thoracic trauma [1]. The aortic isthmus is the most frequent site of traumatic rupture of the thoracic aorta, affecting about 62% of cases [2]. Management of these injuries often involves thoracic endovascular aortic repair (TEVAR), which reduces complications associated with traditional open surgery. The incidence of patients requiring conversion from TEVAR to open thoracic aortic aneurysm repair has been reported at 2.2-7.2% in experienced centers [3]. According to the latest guidelines, TEVAR is currently considered the first-line treatment for traumatic isthmic rupture [4]. In this report, we present a rare case of BTAI involving the aortic isthmus in a 68-year-old female patient.

## Case presentation

A 68-year-old female patient presented to the ED following a pedestrian accident involving a motorcycle. Upon admission, she was conscious, and her vital signs were as follows: blood pressure 138/82 mmHg, heart rate 76 beats per minute, respiratory rate 15 breaths per minute, and oxygen saturation 98% on room air. Her past medical history was suggestive of systemic hypertension, which was optimized with antihypertensives. Clinical examination revealed a symmetrical chest wall, symmetrical respiratory sounds, a soft abdomen, pain in the left distal region below the knee, and pain along the spinal column. The Glasgow Coma Scale was 15/15. X-rays of the chest and vertebral column (lateral view) were normal, but the X-ray of the left lower limb showed a tibia fracture.

A CT scan of the chest and abdomen was performed without the administration of a contrast medium due to a reported allergy to contrast agents. The scan revealed enlargement of the aortic arch up to 4.3 cm, along with linear soft tissue elements of relatively increased density around it. These elements extended and surrounded the descending aorta. Further evaluation was recommended to exclude dissection of the aortic arch. After the CT scan, the patient complained of sudden-onset retrosternal pain. Both ECG and troponin levels were negative. An echocardiogram was subsequently conducted but failed to provide sufficient visualization of both the aortic arch and the descending aorta. However, suspicion arose for an intimal flap, suggesting a potential diagnosis of aortic dissection. As a result, a repeat contrast-enhanced CT (CECT) scan of the chest and abdomen was deemed necessary.

**Figure 1:**
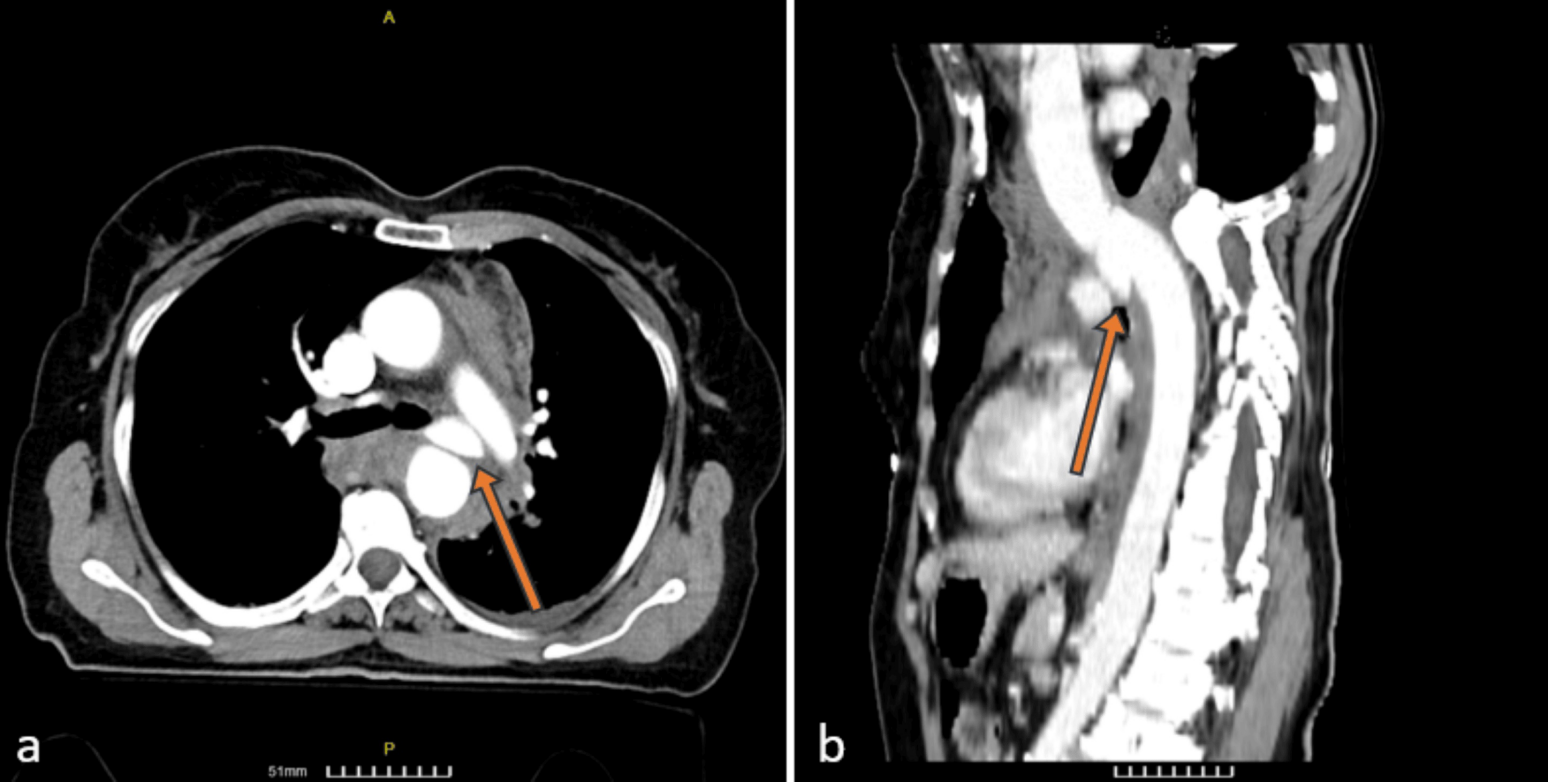
CT before intervention: (a) transverse view and (b) sagittal view The arrow indicates the rupture site at the level of the isthmus.

The contrast allergy prophylaxis protocol was followed for the CECT scan. The updated CT scan revealed an image consistent with acute traumatic injury of the aorta, with a transverse rupture at the level of the isthmus. A pseudoaneurysm and periaortic hematoma were also diagnosed (Figure 1). Following the diagnosis, treatment with beta-blockers and nitrates was initiated to regulate the patient’s arterial blood pressure (121/80 mmHg) and heart rate (70 beats per minute) within the normal range.

**Figure 2:**
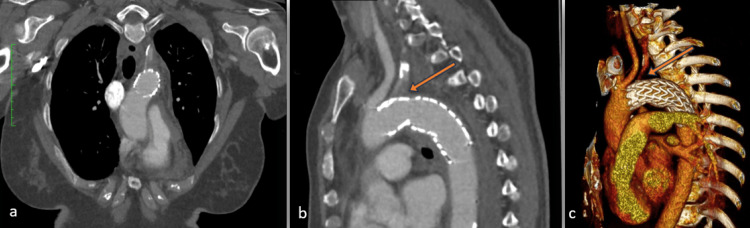
CT after TEVAR implantation: (a) transversal view, (b) sagittal view, and (c) 3D reconstruction The arrow indicates the occlusion of the left subclavian artery. TEVAR, thoracic endovascular aortic repair

Due to the lack of an endovascular surgery department at our hospital, the patient was transferred to the nearest tertiary hospital, located a two-hour drive away. Upon arrival, she was hemodynamically stable and underwent urgent stent implantation (TEVAR) using a C-TAG (GORE) 31-31 × 100 mm endoprosthesis. The occlusion of the left subclavian artery could not be avoided due to the urgent nature of the situation and the presence of a hemothorax resulting from the rupture of the aortic isthmus (Figure 2). An interdisciplinary decision was made to monitor and reassess the need for a possible second-stage carotid-subclavian bypass, depending on the perfusion status of the left arm. However, the perfusion of the left hand remained adequate, and no further vascular surgical intervention was required.

**Figure 3:**
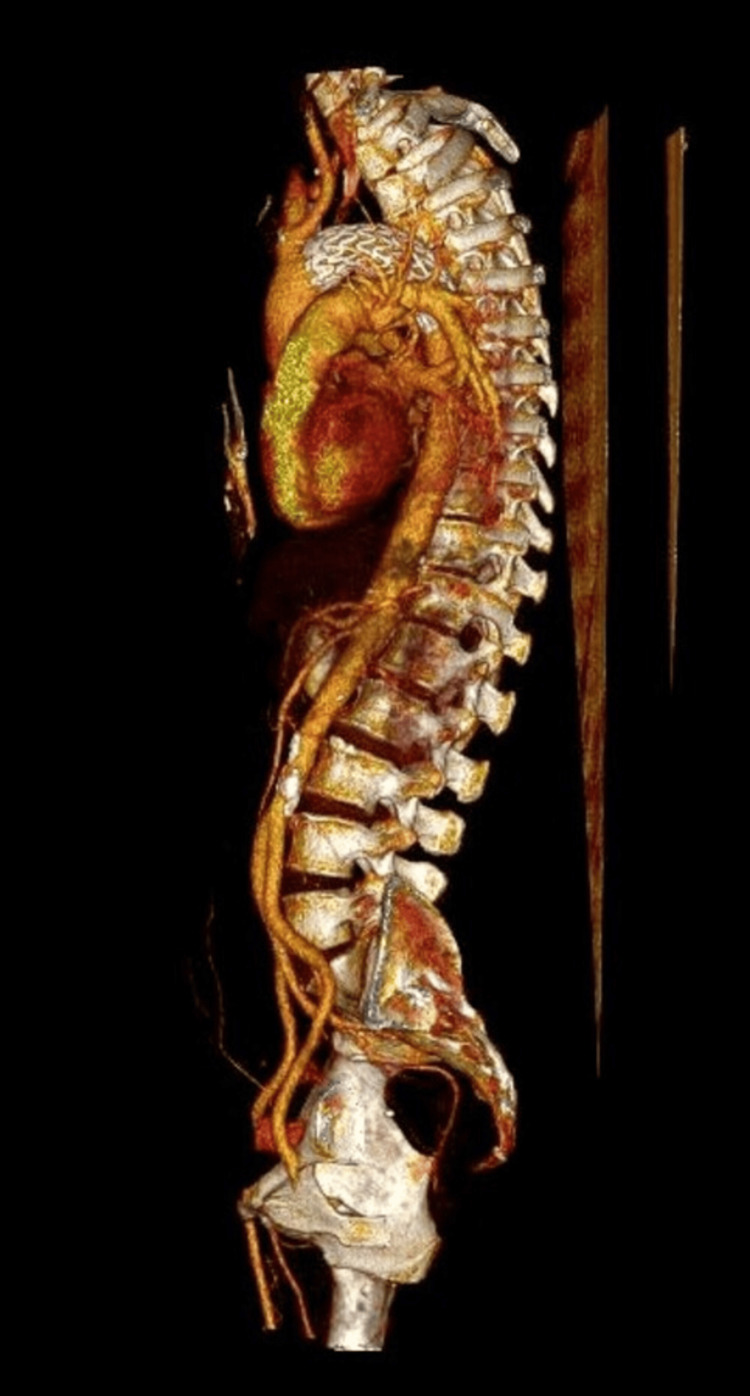
3D reconstruction of the final result

After the successful endovascular repair of the aortic isthmus rupture, the patient was transferred back to our clinic for postoperative care 24 hours later. Postoperative CT angiography showed an excellent result with no signs of endoleak (Figure 3). On the seventh postoperative day, the patient was transferred to the orthopedic ward for further management of the tibia fracture. Following orthopedic treatment, she was discharged and transferred to a rehabilitation center.

## Discussion

The traumatic rupture of the aorta is a severe, life-threatening injury requiring immediate treatment. At least 80% of cases result in death at the scene of the accident, while only 15-20% manage to reach the hospital alive [5]. BTAI is a high-energy trauma that potentially affects multiple aortic zones. The mechanism of injury involves a combination of sudden deceleration and traction of the relatively immobile aortic isthmus (zone 3) [6]. As a result, the aortic isthmus is the most common site of rupture in approximately 90% of BTAI cases [7].

BTAI can be systematically classified based on findings from CT scans. This classification categorizes the extent and nature of the injury into four distinct types. Type I BTAI is characterized by an intimal tear, indicating localized damage to the innermost layer of the aortic wall. Type II is identified as an intramural hematoma, involving bleeding into the layers of the aortic wall without an external rupture. Type III represents a more severe condition, known as a pseudoaneurysm, where the injury results in a contained rupture that forms a false aneurysm. Finally, Type IV BTAI is the most critical, indicating a complete rupture of the aorta, which requires immediate intervention due to its life-threatening nature [6]. The present case was classified as a Type III BTAI, with pseudoaneurysm formation and a periaortic hematoma observed on the CECT scan. This classification was instrumental in guiding the decision to perform TEVAR, the recommended treatment for Type III injuries.

Immediate diagnosis of BTAI is crucial for patient survival, as it is associated with high mortality rates. In hemodynamically stable patients, CECT is the preferred imaging modality for diagnosis. In hemodynamically unstable patients requiring immediate imaging, when CECT is unavailable, transesophageal echocardiography (TEE) is recommended, as it is immediately available and can be performed quickly. However, current guidelines recommend CECT for diagnosing BTAI due to its speed, accuracy, and ability to detect other thoracic injuries [8]. In this case, the CECT scan played a crucial role in diagnosing the rupture and determining its severity, as the TEE did not provide sufficient insight into the underlying pathology.

In recent decades, the use of endovascular stent grafts has significantly improved survival rates and is now considered the preferred treatment for BTAIs whenever possible. However, there are cases in which endovascular repair is not feasible, such as when there is unfavorable anatomy or additional intrathoracic injuries. In these cases, open surgical repair is used as an alternative approach [9].

The management approach for BTAI is determined by the patient’s clinical presentation, hemodynamic status, and BTAI type. Initial management includes the administration of beta-blockers or negative inotropic agents to control systolic blood pressure (preferably <100 mmHg) and heart rate (<100 beats per minute). Additional vasodilators may be used if this therapy is insufficient [9].

Type I BTAIs are typically managed conservatively, while Type II BTAIs are considered in the “gray zone,” initially managed conservatively with close follow-up. If deterioration occurs during follow-up, intervention is necessary for repair. Type III BTAIs are treated with TEVAR, and Type IV BTAIs generally do not survive until reaching the hospital. Those who do reach the hospital with Type IV BTAIs require immediate intervention with TEVAR, provided there is suitable anatomy [1,6]. In cases of multiple life-threatening injuries, it is recommended to prioritize the management of these injuries initially and defer intervention for aortic injury to a secondary phase (after 24 hours). This strategy has been shown to reduce morbidity and mortality rates [8].

Endovascular repair with TEVAR is considered first-line therapy [10]. Unlike conventional open repair, endovascular treatment avoids complications associated with thoracotomy, aortic cross-clamping, and cardiopulmonary bypass. Despite advancements in techniques and devices over the past two decades, common TEVAR complications remain largely unchanged, including spinal cord ischemia, stroke, endoleaks, access site complications, guidewire injuries, retrograde dissections, renal injury, unintentional great vessel coverage, aortoesophageal and aortobronchial fistulas, and device failure. Endovascular repair is indicated for both hemodynamically stable and unstable patients, making it suitable for those requiring immediate treatment [4].

In up to 50% of patients with aortic isthmus rupture, the endovascular graft can occlude the left subclavian artery [4]. In these cases, occlusion and bypass of the left subclavian artery may be necessary to ensure a proximal endograft seal and avoid ischemic complications in the left arm or vertebrobasilar system [11]. This intervention is often required to reduce the risk of ischemic events [9]. Revascularization of the left arm can occur simultaneously with the TEVAR procedure or in a second phase, depending on the perfusion condition of the arm. The decision to monitor left arm perfusion rather than proceed immediately with bypass surgery reflects the clinical judgment required in TEVAR cases. In this case, the left arm perfusion remained adequate, avoiding the need for additional intervention and further reducing the patient’s overall risk.

TEVAR offers significant advantages in terms of mortality, renal failure, spinal cord ischemia, graft infection, and hospital stay duration, with mortality rates ranging from 7% to 9% [4,9]. A cohort study by Cheng et al. found that patients treated with endovascular repair had better long-term survival compared to those treated with conventional surgery (88.9% vs. 71.9% at one year, 88.9% vs. 68.2% at three years, and 88.9% vs. 65.1% at five years) [12]. However, long-term outcomes of TEVAR in BTAI patients are limited, and regular follow-up is necessary [9].

## Conclusions

This case highlights the critical importance of prompt diagnosis and timely intervention in managing BTAI, particularly in cases of traumatic rupture at the aortic isthmus. Despite the rarity of this condition, the successful outcome in this patient underscores the efficacy of endovascular repair (TEVAR) as a treatment modality, even in complex cases involving additional injuries or anatomical challenges. The use of TEVAR enabled rapid stabilization and minimized complications, such as those commonly associated with open surgical repair. While this article emphasizes the wide range of complications and limitations associated with TEVAR, it is also important to reflect on the historical progress in aortic aneurysm repair and recognize the advancements in both endovascular and open surgical techniques. As long-term follow-up data from TEVAR become available in the next decade, clinicians and engineers will continue to refine endovascular technology and expand its applications.

This case also demonstrates the need for a multidisciplinary approach to managing BTAI, particularly in hospitals lacking specialized endovascular surgery departments. The ability to safely transfer hemodynamically stable patients to tertiary centers for definitive treatment is crucial. Furthermore, the decision to defer revascularization of the left subclavian artery in this case emphasizes the importance of individualized patient management, with clinical judgment guiding subsequent interventions based on the patient’s perfusion status. While TEVAR has become the standard of care for BTAI, this case highlights the importance of continued follow-up to monitor long-term outcomes, particularly in complex cases. Further studies are needed to assess the durability of endovascular repair in life-threatening conditions such as BTAI.
